# Dietary Energy Level Impacts the Performance of Donkeys by Manipulating the Gut Microbiome and Metabolome

**DOI:** 10.3389/fvets.2021.694357

**Published:** 2021-10-08

**Authors:** Chongyu Zhang, Chen Zhang, Yunpeng Wang, Meiyu Du, Guiguo Zhang, Yunkyoung Lee

**Affiliations:** ^1^College of Animal Sciences and Technology, Shandong Provincial Key Laboratory of Animal Biotechnology and Disease Control and Prevention, Shandong Agricultural University, Tai'an, China; ^2^Interdisciplinary Graduate Program in Advanced Convergence Technology and Science, Department of Food Science and Nutrition, Jeju National University, Jeju city, South Korea

**Keywords:** digestible energy, growth performance, microbiome, metabolome, donkey

## Abstract

Considerable evidence suggests that dietary energy levels and gut microbiota are pivotal for animal health and productivity. However, little information exists about the correlations among dietary energy level, performance, and the gut microbiota and metabolome of donkeys. The objective of this study was to investigate the mechanisms by which dietary energy content dictates the growth performance by modulating the intestinal microbiome and metabolome of donkeys. Thirty-six nine-month-old male Dezhou donkeys with similar body weights were randomly assigned to two groups fed low- or high-energy diets (LE or HE). The results showed that donkeys fed HE had increased (*p* < 0.05) the average daily gain (ADG) and feed efficiency (G/F) compared with those that received LE diet. The gut microbiota in both groups was dominated by the phyla Firmicutes and Bacteroidetes regardless of the dietary energy level. However, feeding HE to donkeys significantly decreased (*p* < 0.05) the ratio of Firmicutes to Bacteroidetes (F/B). Compared to the LE group, feeding HE specifically increased the abundances of *unidentified_Prevotellaceae* (*p* = 0.02) while decreasing the richness of *unidentified_Ruminococcaceae* (*p* = 0.05). Compared to the LE group, feeding the HE diet significantly (*p* < 0.05) upregulated certain metabolic pathways involving the aspartate metabolism and the urea cycle. In addition, the increased bacteria and metabolites in the HE-fed group exhibited a positive correlation with improved growth performance of donkeys. Taken together, feeding the HE diet increased the richness of Prevotellaceae and upregulated growth-related metabolic pathways, which may have contributed to the ameliorated growth performance of donkeys. Thus, it is a recommendable dietary strategy to feed HE diets to fattening donkeys for superior product performance and feed efficiency.

## Introduction

The Dezhou donkey (*Equus asinus*) is an excellent indigenous herbivorous domestic animal in China, with superior productivity and high forage efficiency (1). Donkey meat is considered as a high-quality food with lower fat and cholesterol but provides abundant unsaturated fatty acids and protein content (2, 3). Additionally, donkey-hide gelatin is a traditional beneficial food with a broad range of functions such as improving immunity and disease resistance. Currently, donkey breeding has become a vital industry in livestock husbandry in China. However, there are no nutritional recommendations for donkeys worldwide; therefore, it is of great significance to explore proper dietary strategies to improve the production efficiency parameters in donkeys.

The gut microbiota is the key mediator through which the diet impacts the host health (4) and productivity (5). Additionally, the dietary energy level is considered to be a pivotal factor that impacts the growth performance and health condition of animals (6), and the richness and compositions of the microbial community residing in the digestible tract are tightly correlated with the energy harvest efficiency of the host (7). It has been documented that the dietary energy level could profoundly affect the gut microbiota and thus result in the changes in nutrient digestibility, microbial protein synthesis, and milk production parameters by intervening in the ruminal microbial community (6). The structure of the ruminal microbiome determines the ability to extract energy from feed ingredients (7, 8). However, how the dietary energy level affects the gut microbiota and growth performance of donkeys remains unclear.

The gut microbiota can ferment dietary ingredients to produce many kinds of metabolites that regulate certain metabolic pathways and impact the physiological and pathological status of the host (9). Short-chain fatty acids (SCFAs) are one of the most important bacterial metabolites. They could act as a direct energy source for intestinal cells, activate intestinal immunity, and modulate animal feed intake (10). Furthermore, SCFAs production from the microbial digestion of dietary fibers provides a substantial portion of the daily energy requirements of horses or donkeys (11). Fecal metabolites can reflect the results of nutrient digestion and absorption by both the gut bacteria and the host gastrointestinal tract, providing a better explanation for the effects of the host–microbiota and metabolome interactions on growth performance (12). Thus, metabolomics application would greatly extend our understanding of how different dietary energy levels affect the metabolism. Karisa et al. (13) and Weikard et al. (14) stated that metabolomics can be used to predict feed efficiency (G/F), average daily gain (ADG), average daily feed intake (ADFI), and dry matter intake. However, current knowledge about the relationships between dietary energy levels and bacterial communities or metabolite profiles in the Dezhou donkeys is limited, and such insights are crucial to the development of technologies that support modern donkey husbandry, providing technical guidance for healthy donkey breeding.

In the current study, it was hypothesized that variation in dietary level would impact the growth performance of donkeys by influencing the intestinal microbiota and metabolome. The objectives of this study was to investigate the effect of dietary energy level on the intestinal microbiome, metabolome, and performance of donkeys, and the relationships among them, thus clarifying whether dietary energy level regulates performance by interfering with the intestinal microbiome and metabolome.

## Materials and Methods

All procedures involving animal care and use were in strict accordance with the animal care and use protocol approved by the Shandong Agricultural University Animal Nutrition Research Institute (Protocol No. S20200068).

### Experimental Design, Animals, and Feeding Management

Thirty-six healthy nine-month-old male Dezhou donkeys with similar body weights (126.5 ± 3 kg) were randomly allocated into six pens with six donkeys in each pen (3 × 10 m). The pens were randomly assigned to two dietary treatments with three replicates per treatment. One treatment was fed a control diet formulated following the feeding standard for Dezhou donkeys in China (DB 37/T 3605−2019) (15) (denoted as the LE group), and the other treatment group received a high digestible energy (DE) diet (denoted as the HE group). The diet compositions and nutritional contents are shown in Table 1.

**Table 1:** Experimental diet composition and nutrient levels.

**Ingredients, %**	**Treatments** ^**a**^	
	**LE**	**HE**
Corn	11.00	44.40
Soybean meal	9.00	13.00
Wheat bran	13.00	6.00
Rice bran meal	13.00	6.00
Wheat flour	8.00	5.00
Rice husk	8.00	5.00
Peanut vine	18.00	9.00
Alfalfa	18.00	9.00
CaHPO_4_	0.25	0.25
Limestone	0.60	1.2
Lysine	0.30	0.30
Met	0.05	0.05
NaHCO_3_	0.30	0.30
NaCl	0.40	0.40
Premix^b^	0.45	0.45
Total	100	100
**Nutrient content** ^c^	
Digestible energy (DE), MJ/kg	10.43	11.90
Crude protein, %	15.00	14.74
Ether extract	2.00	3.00
Neutral detergent fiber, %	33.13	22.92
Acid detergent fiber, %	22.14	11.92
Acid detergent lignin, %	4.43	3.06
Calcium, %	0.71	0.72
Phosphorus, %	0.49	0.41

a *LE, low digestible energy diet; HE, high digestible energy diet*.

b *Supplied per kg of total mixed ration: Vitamins A, 5,000 IU; D, 240 IU; E, 30 IU; K3, 3 mg; B1, 3 mg; B2, 8 mg; B3, 34 mg, B5, 10.8 mg; B6, 4 mg; B7, 0.13 mg; B12, 0.02 mg; Fe, 50 mg; Cu, 8 mg; Zn, 50 mg; Mn, 12.5 mg; Se, 0.20 mg; I, 1 mg*.

c *All items were measured values except DE*.

This experiment consisted of a 20-day adaptation period and a 40-day fattening period for sample collection. Sufficient diets were made in one batch to prevent any batch effect on dietary treatments. Throughout the experimental period, all donkeys had free access to the assigned diets, which were offered two times a day (at 0600 and 1800 h). Furthermore, all the animals had free access to freshwater. The proximate components of feeds were analyzed according to the Association of Official Analytical Chemists (AOAC) (16), and the contents of neutral detergent fiber and acid detergent fiber were determined by the method of Soest et al. (17). Throughout the experimental period, donkeys in each pen were weighed on days 0, 20, and 40 before the morning feeding to determine the ADG. The daily feed offered, orts, and spillages were collected and weighed daily to determine the ADFI. Feed efficiency was expressed as ADG/ADFI (G/F).

### Preparation of Pelleted Total Mixed Ration

The forages for the diets were ground to pass through a 3-mm screen, and all concentrates were ground to pass through a 1.5-mm screen. After mixing, the diet was pelleted at 50–60°C (conditioning temperature) with a compression ratio of 10:1 to form a cylindrical shape (pellet diameter = 6 mm; length = 10–15 mm) using a pelleting machine (18).

### Determination of the Microbiome and Metabolome in Rectum Digesta

At the end of the feeding period, digesta in the rectum were collected from six donkeys within each treatment. Samples were stored in 5-ml frozen pipes and were immediately flash frozen in liquid nitrogen until analysis. The microbiome was determined by 16S rDNA amplicon sequencing. DNA was extracted from rectum digesta samples using the cetyltrimethyl ammonium bromide (CTAB)/sodium dodecyl sulfate (SDS) method, and PCR products were purified with a Qiagen Gel Extraction Kit (Qiagen, Germany). Detailed descriptions of microbe determination are provided in Supplementary Material 1. The metabolome was determined by liquid chromatography–tandem mass spectrometry (LC-MS/MS). First, tissues were individually ground, incubated, and centrifuged. Then, the supernatant was injected into the LC-MS/MS system for analysis, i.e., a Vanquish ultrahigh-performance LC system (Thermo Fisher, Waltham, MA, USA) coupled with an Orbitrap Q Exactive series mass spectrometer (Thermo Fisher). The raw data files generated by UHPLC-MS/MS were processed using the Compound Discoverer 3.1 (CD3.1, Thermo Fisher) to perform peak alignment, peak picking, and quantitation for each metabolite. Detailed descriptions of the metabolome determination are stated in Supplementary Material 2. All the raw data involved in the present study were deposited in the National Center for Biotechnology Information (NCBI) Sequence Read Archive (SRA) under accession number PRJNA722156.

### Statistical Analyses

In this study, the pen was the experimental unit for growth performance measurements (*n* = 3) and microbiota analysis (*n* = 6). ADFI, ADG, and G/F were analyzed in SAS version 9.0 (SAS Institute Inc., Cary, NC, USA). Linear discriminant analysis (LDA) effect size analysis of ruminal microbiota changes was conducted using the online procedure of Galax (http://huttenhower.sph.harvard.edu/galaxy/). Differences were declared to be statistically significant when *p* < 0.05.

## Results

### Growth Performance of Donkeys

The growth performance results of donkeys in different stages are presented in Table 2. No significant difference (*p* = 0.194) was observed for the initial body weight between the LE and HE groups. However, the donkeys fed HE had significantly improved (*p* < 0.05) body weight (BW) and ADG throughout the experiment. In addition, feeding HE increased (*p* < 0.05) the G/F ratio of donkeys in the 40-day experimental period.

**Table 2:** Effects of diets with different energy levels on donkey production performance.

**Items**	**Treatments^a^**		**SEM**	***p*** **-value**
	**LE**	**HE**		
BW^b^(kg)	
Day 0	125.25	128.38	1.070	0.194
Day 40	153.5	160.25	0.944	0.012
Period (0–40 days)
ADG (g/day)	706	797	10.476	0.005
ADFI (g/day)	4,337	4,262	19.582	0.205
G/F	0.16	0.19	0.125	0.003

a *LE, low digestible energy diet; HE, high digestible energy diet*.

b *BW, body weight; ADG, average daily gain; ADFI, average daily feed intake; G/F, feed conversion ratio*.

### Profile and Characteristics of Microbiota in the Rectum

**Figure 1:**
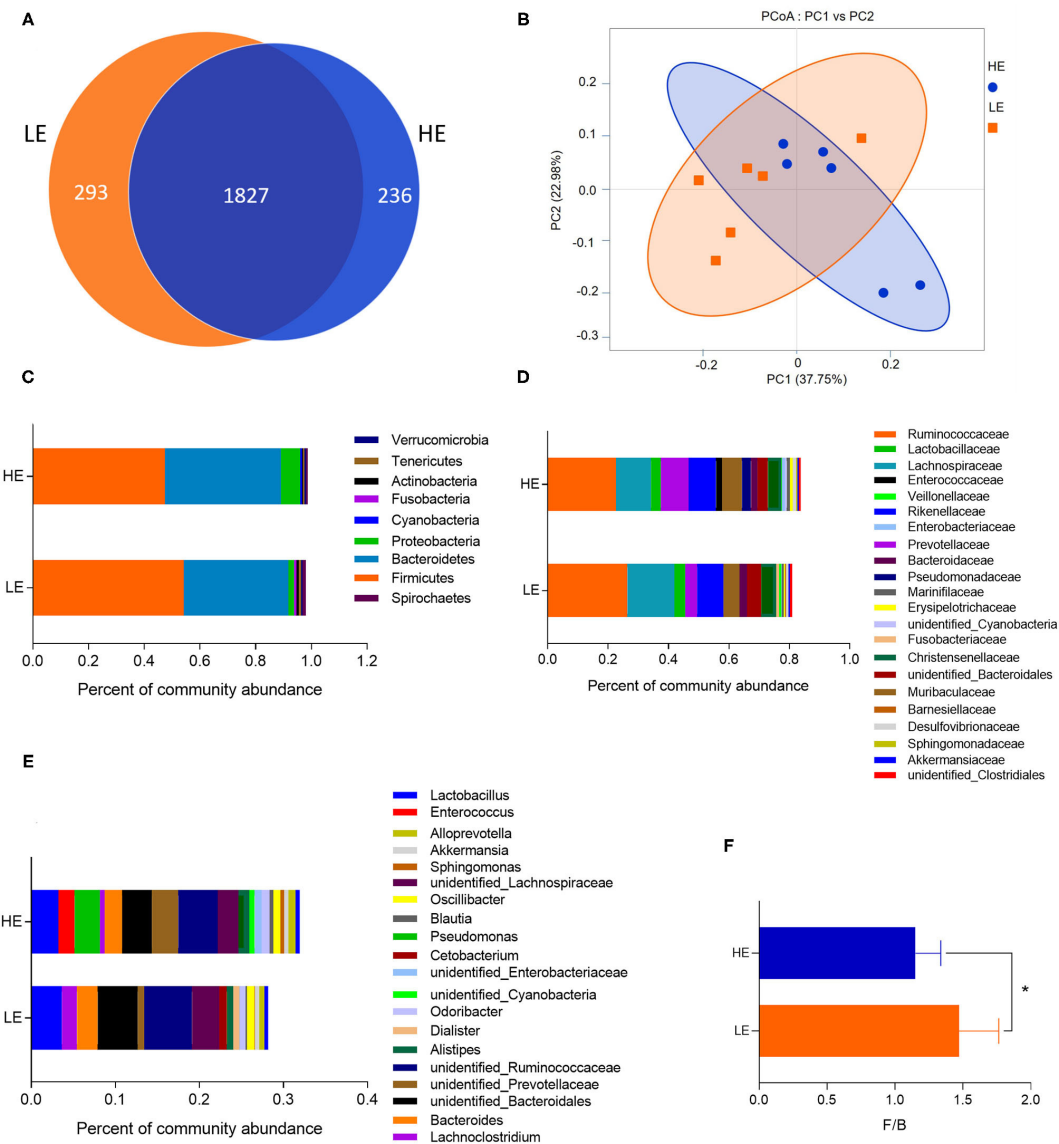
The OTU numbers, beta diversity (PCoA plot), and the relative abundances at the phylum, family, and genus levels of the rectal microbiota of donkeys fed LE or HE diet. **(A)** Venn diagram of OTUs in the rectal microbiota; **(B)** principal coordinate analysis (PCoA). The relative abundances of bacteria on the right in each group at the **(C)** phylum, **(D)** family, and **(E)** genus levels. **(F)** The ratio of Firmicutes and Bacteroidetes. LE and HE represent samples collected in the rectum from donkeys fed LE or HE diet. * means significant difference between the HE and LE group (*p* < 0.05). Only microbes that have a mean relative abundance of more than 0.5% are displayed.

The microbiota of rectum digesta was analyzed in the two dietary groups by sequencing the bacterial 16S rDNA V3 + V4 region. High-throughput pyrosequencing of the samples (*n* = 6/treatment) generated a total of 467,846 and 434,240 raw reads in the LE and HE groups, respectively. After removing low-quality sequences, 436,323 and 401,869 total tags were obtained in the rectum contents of the LE and HE groups, respectively. Considering 97% sequence similarity, a total of 2,120 operational taxonomic units (OTUs) were identified in the LE group, which were assigned to 25 phyla, 39 classes, 82 orders, 141 families, and 247 genera. Meanwhile, 2,063 OTUs were obtained in the HE groups, which belonged to 25 phyla, 37 classes, 70 orders, 127 families, and 239 genera. There were 1,827 OTUs shared by both experimental groups (Figure 1A).

The sequencing depth almost reflected the total microbial species richness (Goods coverage > 99%). Furthermore, no significant differences were observed in the alpha-diversity indices (the Shannon, Simpson, ACE, PD_whole_tree curves, and Chao1) between groups (Table 3). Principle coordinates analysis (PCoA) based on the weighted UniFrac distance revealed no completely separated sample distribution, suggesting a similar structure of the microbial community between the HE and LE groups (Figure 1B).

**Table 3:** Alpha-diversity indices of the rectal microbiota of donkeys.

**Group^a^**	**Coverage %**	**Richness estimator**			**Diversity index**	
		**Chao1**	**ACE**	**PD_whole_tree**	**Shannon**	**Simpson**
LE	>99	1,282.34 ± 103.21	1,301.82 ± 98.15	89.12 ± 10.23	7.82 ± 0.35	0.99 ± 0.01
HE	>99	1,251.01 ± 38.41	1,271.98 ± 41.49	85.31 ± 2.68	7.61 ± 0.37	0.98 ± 0.01

a *low digestible energy diet; HE, high digestible energy diet*.

At the phylum, family, and genus levels, the relative abundance of microbiota with over 0.5% in the rectum was analyzed (Figures 1C–E, Supplementary Tables 1–3). The phyla *Firmicutes* and *Bacteroidetes* dominated the rectal microbiota in both groups, with 54.18 and 37.58% abundance in the LE group and 47.45 and 41.61% abundance in the HE group, respectively (Figure 1C). In addition, the ratio of Firmicutes to Bacteroides in the LE group was greater than that in the HE group (Figure 1F). At the family level, 22 families were identified with the relative abundance of more than 0.5%. The dominant families were Ruminococcaceae, Lachnospiraceae, Prevotellaceae, and Rikenellaceae, with richness of 26.44, 15.45, 3.97, and 8.71% in the LE group and 22.56, 11.65, 9.13, and 9.19% in the HE group, respectively (Figure 1D). Moreover, 21 genera showed relative abundances over 0.5%, and the predominant genera were *unidentified_Ruminococcaceae, unidentified_Bacteroidales, Lactobacillus, unidentified_Lachnospiraceae, Bacteroides*, and *unidentified_Prevotellaceae* (Figure 1E).

### Discrepant Bacteria in the Rectal Microbiota of Donkeys Fed LE or HE

**Figure 2:**
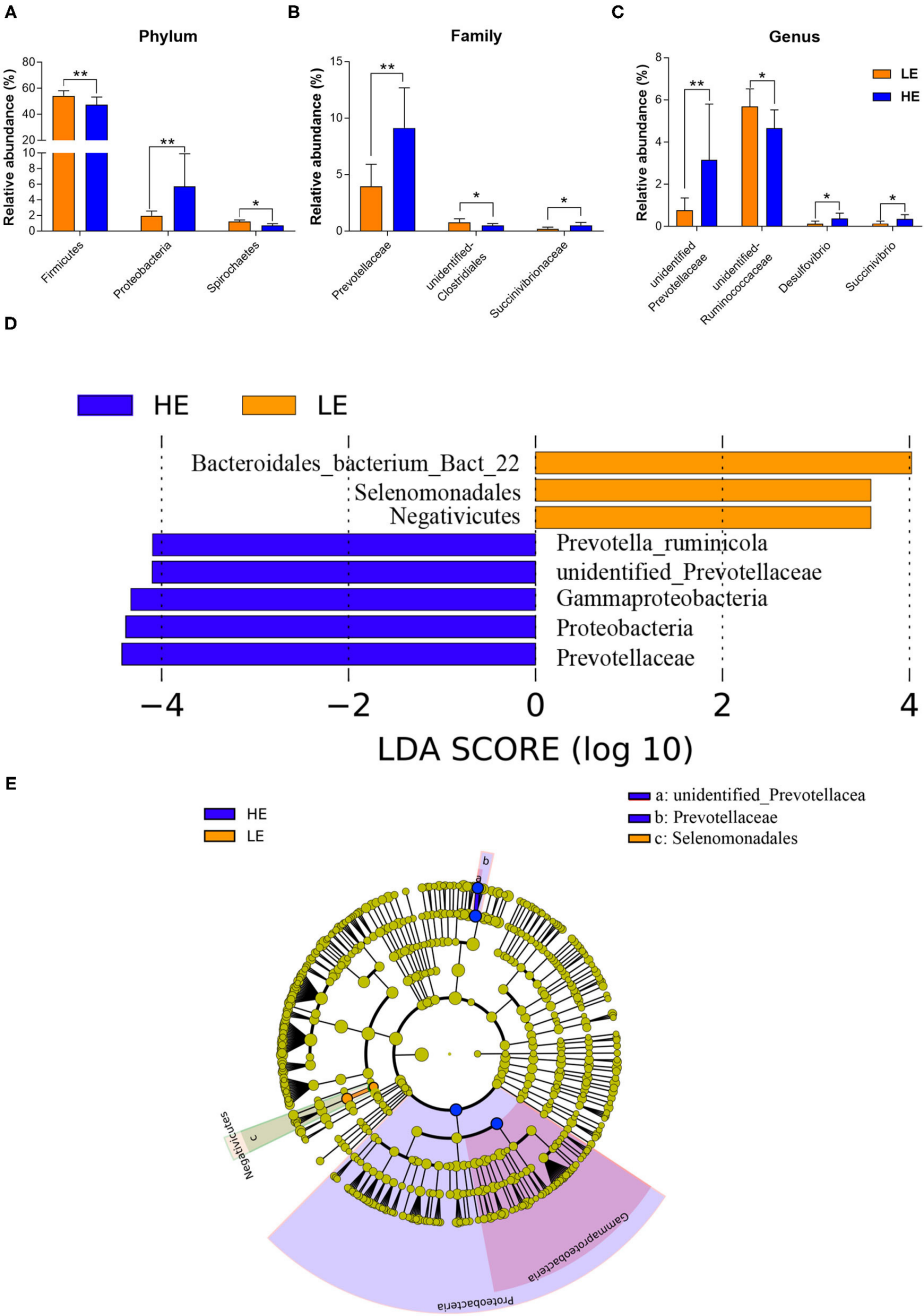
The relative abundances of rectal microbiota that was significantly different between the LE and HE groups at the **(A)** phylum, **(B)** family, and **(C)** genus levels. Only microbes that had a relative abundance of more than 0.1% were compared. Linear discriminant analysis (LDA) value distributed histogram and cladogram of different microorganisms (LDA score > 3.5). **(D)** Linear discriminant analysis (LDA) value distributed histogram. **(E)** Cladogram constructed to visualize the microbial community relative abundance data at rectum samples between the LE and HE groups. Difference was declared to be statistically significant when *P < 0.05, **P < 0.01.

The alterations in the microbial communities between the HE and LE groups at the phylum, family, and genus levels are shown in Figure 2. At the phylum level (Figure 2A; Supplementary Table 1), the relative abundances of Firmicutes, Spirochaetes, and Elusimicrobia in the LE group were significantly higher (*p* < 0.05) than those in the HE group, while feeding HE increased the abundance of Proteobacteria (*p* < 0.05). At the family level, the relative abundances of *unidentified_Clostridiales* (*p* = 0.042) and *unidentified_Elusimicrobia* (*p* < 0.05) were lower in the HE group than those in the LE group. However, the relative abundance of Prevotellaceae and Succinivibrionaceae in the HE group were higher (*p* < 0.05) than those in the LE group (Figure 2B; Supplementary Table 2). At the genus level, the abundances of *unidentified_Prevotellaceae* (*p* = 0.023), *Desulfovibrio* (*P* = 0.026), and *Succinivibrio* (*p* = 0.04) in the HE group tended to be higher than those in the LE group. In contrast, the relative abundances of *unidentified_Ruminococcaceae* and *unidentified_Elusimicrobia* (*p* < 0.05) were higher in the LE group than those in the HE group (Figure 2C; Supplementary Table 3). Similarly, the linear discriminant analysis (LDA) effect size (LEfSe) analysis indicated that feeding HE to donkeys increased the abundance of *Prevotella_ruminicola, unidentified_Prevotellaceae*, Gammaproteobacteria, Proteobacteria, and Prevotellaceae in the rectum while decreasing the richness of *Bacteroidales_bacterium_Bact_22*, Selenomonadales, and Negativicutes compared to those in the LE group (Figures 2D,E). Cladograms were constructed to displayed the phylogenetic distributions of discrepant bacteria in the HE or LE groups (Figure 2E).

### Profile of Rectum Metabolites and Enrichment of Metabolic Pathways

**Figure 3:**
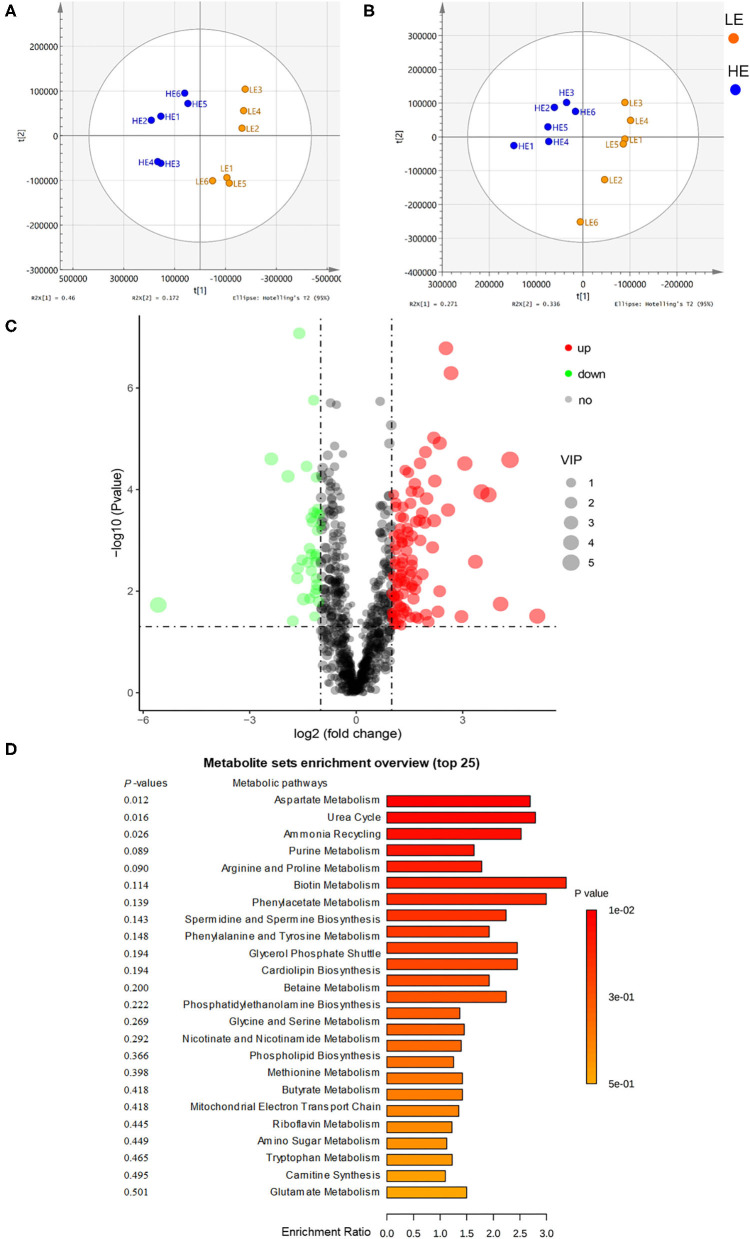
Orthogonal partial least squares discriminant analysis (OPLS-DA) plot of rectal metabolites in comparisons of the LE and HE groups following **(A)** positive ion electrospray ionization (ESI+) and **(B)** negative ion electrospray ionization (ESI–). **(C)** Identification of the differentially abundant metabolites between the LE and HE groups. Red represents an upregulation, while green represents downregulation; blue represents no change. **(D)** Enrichment analysis of metabolic pathways.

The rectum metabolites of donkeys fed with LE and HE were analyzed by a non-targeted LC-MS/MS metabolomics platform, and 999 metabolites (positive and negative ions) were detected in the two groups. Among them, 137 differentiated (log2 fold change > 1.2, *p* < 0.05, variable importance in the projection, VIP >1) metabolites were identified (Figure 3C; Supplementary Tables 4, 5). To compare the distribution of the rectum metabolites of the two groups, the orthogonal projections to latent structures discrimination analysis (OPLS-DA) was conducted (Figures 3A,B). The results displayed a completely separated clustering between the HE and LE groups, suggesting that fecal metabolites were typically differentiated by the energy level of the diets. Compared to the LE group, feeding HE increased (*p* < 0.05) the concentrations of L-aspartic acid, ornithine, L-glutamine, L-phenylalanine, L-serine, methionine, lysine, L-isoleucine, and N-acetylaspartic acid and decreased (*p* < 0.05) the concentrations of phenylpyruvic acid and argininosuccinic acid in the gut content. Additionally, by comparing with the Small Molecule Pathway Database (SMPDB), 137 differential metabolites were allocated into 28 metabolic pathways involving the growth-related essential amino acids metabolism and energy metabolism (Figure 3D). Of them, arginine biosynthesis; phenylalanine, tyrosine, and tryptophan biosynthesis; alanine, aspartate, and glutamate metabolism; and phenylalanine metabolism were significantly (*p* < 0.05) altered by different dietary energy contents.

### Correlations Between Donkey Performance, Rectal Differentiated Bacteria, Metabolites, and Modified Metabolic Pathways

**Figure 4:**
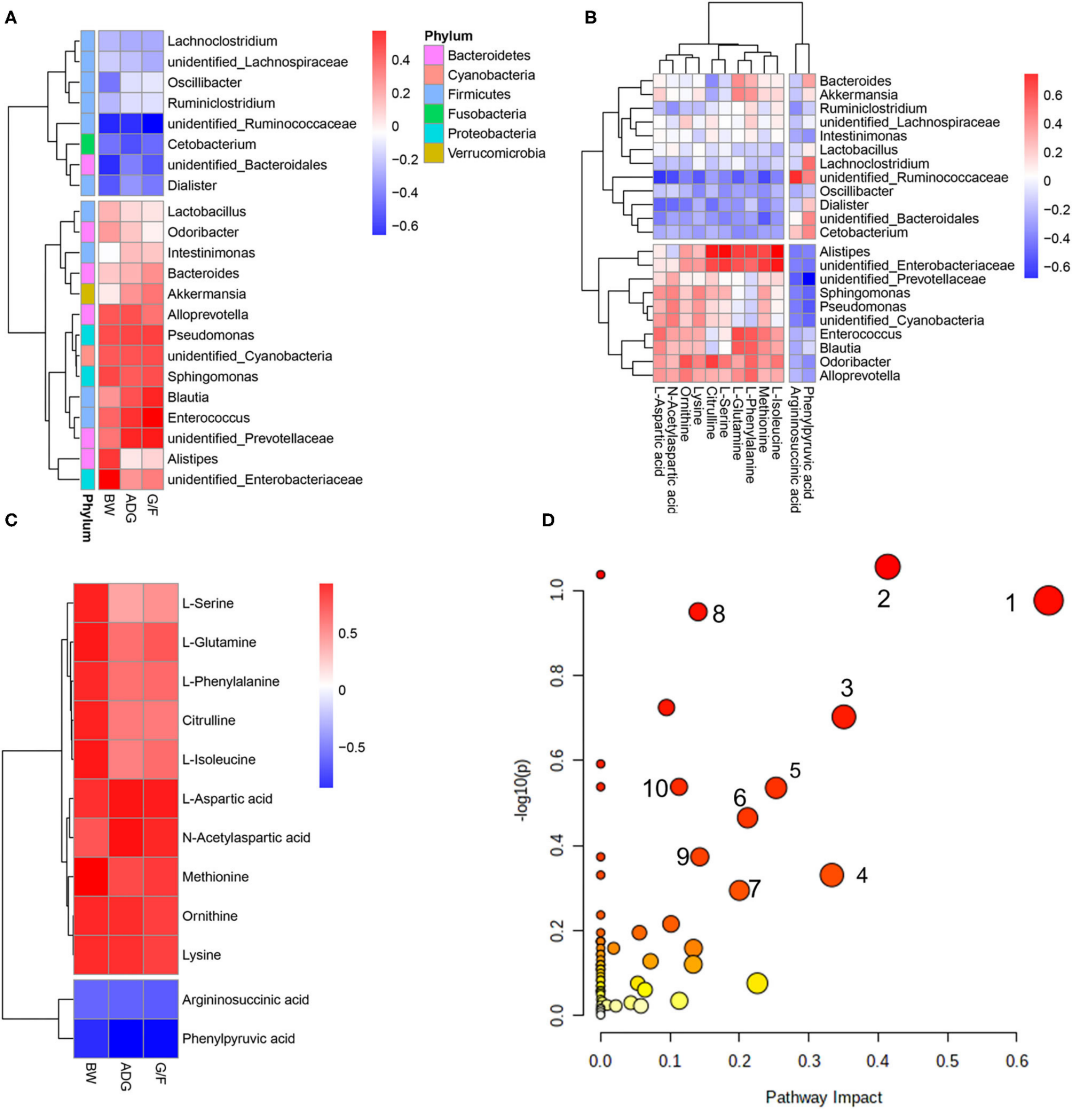
Correlations between **(A)** bacteria in which the relative abundance was more than 0.5% and performance parameters, **(B)** differential metabolites and bacteria, and **(C)** differential metabolites and pathways that differed significantly (*p* < 0.05) and performance parameters. Each row in the graph represents a metabolite, each column represents a performance parameter, and each lattice represents a Pearson correlation coefficient. Red represents a positive correlation, while blue represents a negative correlation. **(D)** Pathway analysis of differential metabolites. The manipulated metabolic pathways are based on the analysis of differentiated ruminal metabolites of donkeys fed HE or LE diets following the Bos Taurus KEGG pathway database. The metabolome view shows all matched pathways according to the *p*-values from the pathway enrichment analysis and impact values from the topology analysis. The node colors varied from yellow to red, indicating that the metabolites have in the data with different levels of significance. 1, Aspartate metabolism; 2, urea cycle; 3, cardiolipin biosynthesis; 4, glycerol phosphate shuttle; 5, glycerolipid metabolism; 6, arginine and proline metabolism; 7, malate–aspartate shuttle; 8, ammonia recycling; 9, amino sugar metabolism; 10, histidine metabolism.

A Pearson's correlation analysis was performed to evaluate the correlation between performance, rectal differentiated bacteria, and metabolites (Figure 4). The associations between microbes and metabolites provided a comprehensive understanding of the composition and function of the microbiota.

The predominant genus of *unidentified_Prevotellaceae* was positively correlated with ADG and G/F, while *unidentified_Ruminococcaceae* was negatively correlated with the growth parameters (Figure 4A). *Unidentified_Prevotellaceae*, which showed increased abundance in the HE group, was negatively associated with phenylpyruvic acid and argininosuccinic acid but exerted a positive correlations with L-aspartic acid, ornithine, methionine, lysine, L-isoleucine, and N-acetylaspartic acid (Figure 4B). Furthermore, those metabolites showed positive correlations with BW, ADG, and G/F (Figure 4C). The analysis of metabolic pathways from the differential metabolites revealed that several metabolites concentrations including argininosuccinic acid, citrulline, L-aspartic acid, ornithine, L-glutamine, L-phenylalanine, L-serine, methionine, lysine, L-isoleucine, phenylpyruvic acid, and N-acetylaspartic acid related to arginine biosynthesis; aminoacyl-tRNA biosynthesis; phenylalanine, tyrosine, and tryptophan biosynthesis; and alanine, aspartate, and glutamate metabolism (Figure 4D). In contrast, *unidentified_Ruminococcaceae*, which showed decreased richness in the HE group compared to that in the LE group, was negatively associated with L-aspartic acid, L-glutamine, methionine, lysine, and N-acetylaspartic (those metabolites were positively correlated with ADG and G/F) and positively associated with argininosuccinic acid and phenylpyruvic acid, which negatively correlated to ADG and G/F (Figure 4C). Additionally, *Alistipes* and *unidentified_Enterobacteriaceae* were positively associated with citrulline, L-serine, L-glutamine, L-phenylalanine, methionine, and L-isoleucine. These metabolites including citrulline, L-aspartic acid, L-glutamine, L-phenylalanine, L-serine, methionine, lysine, L-isoleucine, and N-acetylaspartic acid related to aminoacyl-tRNA biosynthesis; phenylalanine, tyrosine, and tryptophan biosynthesis; and alanine, aspartate, and glutamate metabolism (Figure 4D).

## Discussion

The results of this study demonstrated that feeding HE to donkeys enhanced the growth performance in terms of ADG and G/F. Similarly, Ge et al. (19) demonstrated that broilers fed HE had greater G/F than the basal energy groups, while Fang et al. (20) indicated that G/F decreased as the dietary metabolic energy level decreased over the entire experimental period. Moreover, Ahmad et al. (21) found that G/F was significantly higher with an increased dietary energy level. Our findings suggested that the dietary energy content changed the composition and abundance of the gut microbial community, and specifically increased the abundance of *Prevotellaceae*, thus modulating some growth-related metabolic pathways, which contributed to the improved production performance of donkeys.

The structure (composition and richness) and function (metabolic mechanism) of the intestinal microbiome are crucial to animal health and metabolism (22) in a highly dynamic symbiotic relationship. Visconti et al. (23) addressed an intense interplay between the gut microbiome and its host. In this study, alpha-diversity analysis showed no significant (*p* > 0.05) difference in the rectal bacterial community between the two dietary energy levels. Our previous studies have shown that dietary form affected ruminant microbial composition and abundance and thus the performance of lambs (18). This was consistent with the results of this study.

More than 90% of the species in the bacterial community belong to Firmicutes and Bacteroidetes (24). In our study, Firmicutes and Bacteroidetes were the dominant bacteria, agreeing with the observations from previous studies on microbial communities of monogastric herbivorous animals. For example, Su et al. (22) found that the phyla with the greatest abundances were the Firmicutes (55.01%) and Bacteroidetes (24.76%) in horses. Similarly, Liu et al. (25) pointed out that both Firmicutes and Bacteroidetes are abundant (both accounting for >40%) in the hindgut of Dezhou donkeys. Moreover, Zhao et al. (26) demonstrated that *Firmicutes* and *Bacteroidetes* were the most abundant and predominant phyla in horse fecal samples. The F/B ratio is a vital index indicating the composition and richness of the gut microbial community, which exerts a tight linkage to the energy harvest efficiency of ruminants from the diet (7) and the changes in body weight in humans (27). For instance, a high F/B ratio is associated with obesity and the production of SCFAs such as butyrate and propionate (9, 28). In this study, feeding HE to donkeys significantly decreased the ratio of Firmicutes to Bacteroidetes (F/B) with the increased richness of Bacteroidetes, which can degrade dietary carbohydrates and produce the SCFAs (29). SCFAs are the energy source and signaling molecules that regulate the feed intake and immune status of the host (9). Thus, the increased *Bacteroidetes* proliferation due to feeding HE might be a vital contributor to the improved growth performance. Ruminococcaceae is a family in the phylum Firmicutes and can degrade for the degradation of fibrous carbohydrates, and Prevotellaceae is the dominant family in the phylum Bacteroidetes and can degrade the starch (29). In this study, *unidentified_Prevotellaceae* and *unidentified_Ruminococcaceae* in the HE group had significantly higher and lower relative abundances, respectively, than those in the LE group. This could be explained by HE having more starch, as the proportion of corn was higher than that in LE. Sanchez-Tapia et al. (30) stated that *Prevotella* is associated with the intake of carbohydrates and simple sugars. In addition, cellulolytic *Clostridia*, which are ubiquitous in cellulosic anaerobic environments, represent a major paradigm for the efficient biological degradation of cellulosic biomass (31). In our study, Lachnospiraceae and *unidentified_Clostridiales* had greater abundance in LE- than that in HE-fed donkeys, which may be explained by LE having more fiber sources of peanut vine and alfalfa than HE. Lachnospiraceae is the dominant family in the order Clostridiales, within the phylum Firmicutes (32). This belongs to the fibrolytic community and has been associated with the production of butyrate necessary for the health of colonic epithelial tissue (33). *Clostridia* are known as major producers of short-chained fatty acids, which are important energy sources for enterocytes and also exert immunoregulatory functions (34).

The interaction between the gut microbiota diet is a moderator that impacts the host physiology and metabolic processes (4, 35). When the diet provides adequate amounts of protein, the increase in energy levels produced by adding carbohydrates can improve protein synthesis (36). In this study, we found that feeding different energy levels to donkeys significantly altered the concentration of most metabolites associated with protein digestion and absorption and the biosynthesis of amino acids. Aminoacyl-tRNA synthetases are an essential and universally distributed family of enzymes that play a critical role in protein synthesis, pairing tRNAs with their cognate amino acids for decoding mRNAs (37, 38). In our study, L-phenylalanine, L-glutamine, L-aspartate, L-serine, L-methionine, L-lysine, and L-isoleucine were upregulated in the HE group, which indicated that HE can promote the synthesis of protein. Glutamate was the largest contributor to the tricarboxylic acid cycle intermediate fluxes, and its dietary composition altered L-glutamate catabolism (39). Although cysteine and methionine metabolism had no impact (only two hits), the level of L-methionine in HE-fed donkeys was higher (*p* < 0.05) than that in LE-fed donkeys. L-Methionine is a precursor to other sulfur-containing amino acids, and it is the essential and limiting amino acid for donkey growth and production. In addition, L-methionine also plays an important role in intestinal bacterial protein synthesis.

The abundance and composition of intestinal microbiota is believed to be correlated with energy harvesting and performance (40). In this study, changes in intestinal microbial abundance induced by diet resulted in a shifted metabolome of intestinal microbiota, as shown by Pearson's correlation analysis. *Unidentified_Ruminococcaceae* was negatively associated with ornithine, L-phenylalanine, and L-arginine, while it showed a positive correlation with argininosuccinic acid and phenylpyruvic acid. Phenylpyruvic acid and argininosuccinic acid had a negative correlation with donkey parameters, possibly owing to LE altering the arginine biosynthesis pathway and arginine synthesis from argininosuccinic acid, which could cause excessive ammonia in the body that is harmful for growth and health. *Unidentified_Prevotellaceae* was negatively correlated with phenylpyruvic acid and argininosuccinic acid but showed positive correlations with ornithine, L-phenylalanine, and L-arginine, suggesting that higher production of microbiota-derived amino acids may be positively correlated with higher performance in donkeys. Phenylpyruvate is an intermediate product of the conversion from phenylalanine to valine. Accumulation in the body means that the transformation pathway is blocked, which often leads to phenylpyruvuria and valine deficiency. Valine is a precursor to the synthesis of a range of neurotransmitters and hormones in animals, and its lack can lead to poor growth performance. Argininosuccinic acid is an important component involved in the ornithine cycle in the body, and its enrichment (high content) generally indicates that the excretion of ammonia was blocked. High concentrations of arginine succinate and ammonia caused cytotoxicity and reduced energy utilization and causally resulted in the decreased health conditions and productivity (41).

Several studies have found that both positive and negative associations between specific gut bacteria and metabolism and intestinal microbiota are associated with various metabolic pathways, such as lipid metabolism and amino acid synthesis (42, 43). Our metabolome data revealed that different energy diets alter the concentrations of metabolites in the rectum and indicate that rectal metabolism might be linked with microbial activities. Additionally, Koh and Backhed (44) demonstrated that an absence of microbial compositional changes does not necessarily mean an absence of microbial contributions to host metabolism. However, there is not enough evidence to indicate the microbe species and count relation with certain metabolic products in gastrointestinal samples. Thus, additional efforts have to be directed toward increasing our knowledge in terms of causality and mechanisms.

Taken together, compared to the LE group, feeding the HE diet to donkeys characterized the gut microbiome with a low diversity but improved the richness of certain specific efficient bacteria. Moreover, there were tight correlations between the abundance of microbes, contents of metabolites, and performance in the HE group. This revealed that the interaction of dietary energy content and gut microbiota reshaped the exclusive configuration of the microbial community, which underpinned the alteration of the growth-promoting metabolites and metabolic pathways and thus resulted in the increased production performance of donkeys. It has been suggested that increased enrichment of specific microbes and metabolic pathways rather than the greater diversity contributes to the better energy harvest and improved production performance of animals (7, 40). This was consistent with our current results.

The present results supported our hypothesis that feeding a HE diet altered the gut microbiome and metabolome and upregulated the growth-related metabolic pathways, which underlie the increased production performance and feed efficiency. Additionally, this study provided a new viewpoint for understanding the underlying mechanisms by which different dietary energy content impact the growth performance of animals by intervening in the axis of “microbiome–metabolome–phenotypes” to achieve the superior productivities of animals. The findings also suggested that the specific growth-related microbes and metabolites may be the potential targets for modifying the production performance of animals by specific diet consumption, which provided a novel perspective for developing a dietary strategy to improve the production performance of donkeys reared in an intensive feeding system.

## Conclusions

In conclusion, feeding the HE diet improved the growth performance and feed efficiency by increasing the ADG and G/F ratio. Regardless of dietary energy level, the gut microbiota in both groups was dominated by the phyla Firmicutes and Bacteroidetes. However, feeding HE to donkeys significantly decreased (*p* < 0.05) the ratio of Firmicutes to Bacteroidetes (F/B). Additionally, feeding HE diet characterized the rectum microbiome with a decreased alpha-diversity and the relative abundance of *unidentified_Ruminococcaceae* in the rectum, whereas increased abundance of specific microbes involving Fibrobacter, Rikenellaceae, and Veillonellaceae. Meanwhile, feeding HE diets improved the concentrations of L-aspartic acid, ornithine, L-glutamine, L-phenylalanine, L-serine, methionine, lysine, L-isoleucine, and N-acetylaspartic acid in the gut content of donkeys and thus affected some growth-related metabolic pathways mainly involving aspartate metabolism and urea cycle. The increased bacteria and metabolites in the HE group exhibited the positive correlation with the ADG and feed efficiency of donkeys. Thus, the HE diet increased the richness of beneficial bacteria and thus modified the growth-related metabolic pathways, which contributed to the improved performance and feed efficiency of donkeys. These beneficial bacteria and metabolites related to dietary energy concentration are potential targets for regulating growth performance. The present findings also provide an innovative insight for developing the new growth-promoting probiotics and prebiotics.

## Data Availability Statement

The datasets presented in this study can be found in online repositories. The names of the repository/repositories and accession number(s) can be found at: NCBI SRA; PRJNA722156.

## Author Contributions

GZ and YL designed the experiments. CheZ, YW, and MD conducted the experiments. YW and MD performed the determination of samples. ChoZ and CheZ analyzed the data. CheZ, GZ, and YL wrote and revised the manuscript. All authors read and approved the final manuscript.

## Funding

This work was supported by the Funds of Shandong Province Agricultural Major Applied Technology Innovation Project (SD2019XM001), the Donkey Industry Innovation Team (SDAIT-30-02), the Forage Industrial Innovation Team project (SDAIT-23-05), the National Key R&D program of China–Korea cooperative project (2019YFE0107700; NRF-2019K1A3A1A20081146), the National Research Foundation Grant of Korea (NRF-2017R1D1A3B03031665, NRF-2020R1A2C2004144), and the Excellent Seed Project (2019LZGC012 and LZ201712080160).

## Conflict of Interest

The authors declare that the research was conducted in the absence of any commercial or financial relationships that could be construed as a potential conflict of interest.

## Publisher's Note

All claims expressed in this article are solely those of the authors and do not necessarily represent those of their affiliated organizations, or those of the publisher, the editors and the reviewers. Any product that may be evaluated in this article, or claim that may be made by its manufacturer, is not guaranteed or endorsed by the publisher.
