# Supplementary material for: Dietary Energy Level Impacts the Performance of Donkeys by Manipulating the Gut Microbiome and Metabolome
Source: Front Vet Sci. 2021 Oct 8;8:694357. doi: 10.3389/fvets.2021.694357 (PMC8531409; doi:10.3389/fvets.2021.694357)
Supplement: Supplementary file 1 [file Data_Sheet_1.docx]

**Supplementary materials**

**Dietary energy level impacts the performance of donkeys by manipulating the gut microbiome and metabolome**

C.Y. Zhang^1,^ *, C. Zhang^1,^ *, Y.P. Wang^1^, M.Y. Du^1^, G.G. Zhang^1, †^, Y. Lee ^2, †^

^1^College of Animal Sciences and Technology, Shandong Provincial Key Laboratory of Animal Biotechnology and Disease Control and Prevention, Shandong Agricultural University, 61 Daizong Street, Taian City, Shandong Province, 271018, China

^2^Interdisciplinary Graduate Program in Advanced Convergence Technology and Science, Department of Food Science and Nutrition, Jeju National University, Jeju 63243, South Korea

**† Correspondence:**

G.G. Zhang [zhanggg@sdau.edu.cn](mailto:zhanggg@sdau.edu.cn)

Y. Lee [lyk1230@jejunu.ac.kr](mailto:lyk1230@jejunu.ac.kr)

*These authors contributed equally to this study and share the first authorship.

**The determination of rectum digesta microbiome of donkey**

There are four sequential steps for the methods: Extraction of genome DNA, Amplicon Generation, PCR Products quantification and qualification, and Library preparation and sequencing.

**1. Extraction of genome DNA**

Total genome DNA from samples was extracted using CTAB/SDS method. DNA concentration and purity was monitored on 1% agarose gels. According to the concentration, DNA was diluted to 1ng/µL using sterile water.
**2. Amplicon Generation:**

The V3-V4 hypervariable region of 16S rRNA genes were amplified used specific primer (forward primers: 5′TAG-ATACCCSSGTAGTCC3′ and reverse primers: 5′ CTGACGRCRGCCATGC3′) [1]. All PCR reactions were carried out with 15 µL of Phusion® High-Fidelity PCR Master Mix (New England Biolabs); 0.2 µM of forward and reverse primers, and about 10 ng template DNA. Thermal cycling consisted of initial denaturation at 98℃ for 1 min, followed by 30 cycles of denaturation at 98℃ for 10 s, annealing at 50℃ for 30 s, and elongation at 72℃ for 30 s. Finally 72℃ for 5 min.

**3. PCR Products quantification and qualification:**

Mix same volume of 1X loading buffer (contained SYB green) with PCR products and operate electrophoresis on 2% agarose gel for detection. PCR products was mixed in equidensity ratios. Then, mixture PCR products was purified with Qiagen Gel Extraction Kit (Qiagen, Germany).

**4. Library preparation and sequencing:**

Sequencing libraries were generated using TruSeq® DNA PCR-Free Sample Preparation Kit (Illumina, USA) following manufacturer's recommendations and index codes were added. The library quality was assessed on the Qubit 2.0 Fluorometer (Thermo Scientific) and Agilent Bioanalyzer 2100 system. At last, the library was sequenced on an Illumina NovaSeq platform and 250 bp paired-end reads were generated. The raw paired-end reads were assembled into longer sequences and quantitatively filtered by PANDAseq (version 2.9) to remove the low-quality reads with a length of <220 nucleotides (nt) or >500 nt, an average quality score of <20, and sequences containing >3 nitrogenous bases. The high-quality sequences were clustered into OTUs with a 97% similarity using UPARSE (version 7.0) in QIIME (version 1.8), and the chimeric sequences were removed using UCHIME. Taxonomy was assigned to OTUs using the RDP classifier1 against the SILVA 16S rRNA gene database (Release1282), with a confidence threshold of 70%.

**Table S1** relative abundance (%) of bacterial phyla in the rectal microbiota of donkeys receiving Low-energy diet and High-energy diet.

| Taxa | Groups | | |
| --- | --- | --- | --- |
| phylum | LE | HE | *p*-value |
| *Firmicutes* | 54.189 | 47.452 | 0.046 |
| *Bacteroidetes* | 37.577 | 41.611 | 0.155 |
| *Proteobacteria* | 1.971 | 6.893 | 0.098 |
| *Fusobacteria* | 0.982 | 0.013 | 0.355 |
| *Cyanobacteria* | 0.006 | 0.655 | 0.209 |
| *Actinobacteria* | 0.834 | 0.501 | 0.288 |
| *Tenericutes* | 0.751 | 0.365 | 0.162 |
| *Verrucomicrobia* | 0.549 | 0.524 | 0.926 |
| *Spirochaetes* | 1.237 | 0.744 | 0.002 |
| *Acidobacteria* | 0.301 | 0.097 | 0.357 |
| *unidentified_Bacteria* | 0.317 | 0.272 | 0.768 |
| *Elusimicrobia* | 0.366 | 0.141 | 0.016 |
| *Rokubacteria* | 0.156 | 0.047 | 0.330 |
| *Melainabacteria* | 0.134 | 0.174 | 0.630 |
| *Chloroflexi* | 0.139 | 0.064 | 0.313 |
| *Fibrobacteres* | 0.162 | 0.211 | 0.213 |
| *Deferribacteres* | 0.053 | 0.056 | 0.935 |
| *Nitrospirae* | 0.042 | 0.009 | 0.268 |
| *Kiritimatiellaeota* | 0.078 | 0.074 | 0.784 |
| *Synergistetes* | 0.022 | 0.011 | 0.478 |
| *Latescibacteria* | 0.024 | 0.006 | 0.335 |
| *Gemmatimonadetes* | 0.023 | 0.015 | 0.577 |
| *Euryarchaeota* | 0.000 | 0.003 | 0.275 |
| *Lentisphaerae* | 0.007 | 0.005 | 0.441 |
| *Armatimonadetes* | 0.005 | 0.003 | 0.449 |
| *Planctomycetes* | 0.001 | 0.000 | 0.363 |
| *Others* | 0.076 | 0.057 | 0.383 |

The data is the means of 6 replicated samples.

**Table S2** relative abundance (%) of bacterial family in the rectal microbiota of donkeys receiving Low-energy diet and High-energy diet.

| Taxa | Groups | | |
| --- | --- | --- | --- |
| family | LE | HE | *P*-value |
| *Ruminococcaceae* | 26.441 | 22.563 | 0.210 |
| *Lachnospiraceae* | 15.448 | 11.653 | 0.302 |
| *Lactobacillaceae* | 3.624 | 3.233 | 0.904 |
| *Prevotellaceae* | 3.965 | 9.132 | 0.015 |
| *Rikenellaceae* | 8.708 | 9.189 | 0.726 |
| *Enterococcaceae* | 0.031 | 1.943 | 0.345 |
| *Muribaculaceae* | 5.249 | 6.595 | 0.299 |
| *Pseudomonadaceae* | 0.010 | 3.007 | 0.176 |
| *Bacteroidaceae* | 2.463 | 2.082 | 0.823 |
| *unidentified_Bacteroidales* | 4.760 | 3.526 | 0.210 |
| *Christensenellaceae* | 4.867 | 4.486 | 0.367 |
| *Fusobacteriaceae* | 0.982 | 0.013 | 0.355 |
| *Veillonellaceae* | 0.826 | 0.090 | 0.250 |
| *Enterobacteriaceae* | 0.074 | 0.981 | 0.107 |
| *unidentified_Cyanobacteria* | 0.006 | 0.655 | 0.209 |
| *Marinifilaceae* | 0.736 | 1.038 | 0.567 |
| *Erysipelotrichaceae* | 0.371 | 0.961 | 0.166 |
| *Barnesiellaceae* | 0.409 | 0.002 | 0.358 |
| *Desulfovibrionaceae* | 0.662 | 1.003 | 0.374 |
| *Sphingomonadaceae* | 0.065 | 0.542 | 0.174 |
| *Akkermansiaceae* | 0.456 | 0.504 | 0.853 |
| *unidentified_Clostridiales* | 0.782 | 0.509 | 0.092 |
| *Peptostreptococcaceae* | 0.136 | 0.258 | 0.578 |
| *Staphylococcaceae* | 0.009 | 0.200 | 0.365 |
| *Succinivibrionaceae* | 0.202 | 0.516 | 0.028 |
| *Helicobacteraceae* | 0.240 | 0.166 | 0.634 |
| *Acidaminococcaceae* | 0.424 | 0.314 | 0.179 |
| *Burkholderiaceae* | 0.089 | 0.257 | 0.131 |
| *Spirochaetaceae* | 0.392 | 0.317 | 0.195 |
| *unidentified_Elusimicrobia* | 0.297 | 0.077 | 0.021 |
| *Anaeroplasmataceae* | 0.156 | 0.037 | 0.153 |
| *Bifidobacteriaceae* | 0.092 | 0.009 | 0.291 |
| *Xanthobacteraceae* | 0.146 | 0.071 | 0.473 |
| *Flavobacteriaceae* | 0.002 | 0.121 | 0.186 |
| *Eggerthellaceae* | 0.157 | 0.173 | 0.790 |
| *unidentified_Acidobacteria* | 0.100 | 0.032 | 0.362 |
| *Tannerellaceae* | 0.128 | 0.166 | 0.559 |
| *Fibrobacteraceae* | 0.162 | 0.211 | 0.213 |
| *Chthoniobacteraceae* | 0.077 | 0.015 | 0.292 |
| *unidentified_Bacteria* | 0.056 | 0.088 | 0.465 |
| *Beijerinckiaceae* | 0.010 | 0.077 | 0.158 |
| *Gaiellaceae* | 0.056 | 0.016 | 0.349 |
| *Nitrosomonadaceae* | 0.050 | 0.026 | 0.555 |
| *Coriobacteriaceae* | 0.034 | 0.001 | 0.315 |
| *Bacillaceae* | 0.036 | 0.019 | 0.637 |
| *Pyrinomonadaceae* | 0.041 | 0.009 | 0.339 |
| *Deferribacteraceae* | 0.053 | 0.056 | 0.935 |
| *Peptococcaceae* | 0.104 | 0.099 | 0.853 |
| *Rhizobiaceae* | 0.021 | 0.051 | 0.380 |
| *unidentified_Alphaproteobacteria* | 0.052 | 0.014 | 0.222 |
| *Nitrospiraceae* | 0.042 | 0.009 | 0.268 |
| *Elusimicrobiaceae* | 0.069 | 0.064 | 0.820 |
| *Defluviitaleaceae* | 0.059 | 0.033 | 0.194 |
| *Aeromonadaceae* | 0.021 | 0.000 | 0.363 |
| *Microbacteriaceae* | 0.003 | 0.039 | 0.178 |
| *Streptococcaceae* | 0.082 | 0.063 | 0.122 |
| *unidentified_Rhizobiales* | 0.029 | 0.027 | 0.920 |
| *Moraxellaceae* | 0.020 | 0.015 | 0.807 |
| *Desulfarculaceae* | 0.029 | 0.005 | 0.273 |
| *Atopobiaceae* | 0.016 | 0.056 | 0.084 |
| *Caulobacteraceae* | 0.003 | 0.029 | 0.180 |
| *Sphingobacteriaceae* | 0.002 | 0.030 | 0.198 |
| *Mycoplasmataceae* | 0.018 | 0.004 | 0.414 |
| *Rhodanobacteraceae* | 0.020 | 0.008 | 0.492 |
| *Synergistaceae* | 0.022 | 0.011 | 0.478 |
| *Acidothermaceae* | 0.019 | 0.003 | 0.340 |
| *Streptomycetaceae* | 0.020 | 0.006 | 0.353 |
| *Spirosomaceae* | 0.001 | 0.024 | 0.187 |
| *Anaerolineaceae* | 0.022 | 0.033 | 0.329 |
| *unidentified_Gammaproteobacteria* | 0.009 | 0.013 | 0.761 |
| *Micrococcaceae* | 0.027 | 0.018 | 0.497 |
| *Acetobacteraceae* | 0.012 | 0.010 | 0.880 |
| *Mycobacteriaceae* | 0.021 | 0.003 | 0.239 |
| *unidentified_Solibacterales* | 0.021 | 0.007 | 0.348 |
| *Frankiaceae* | 0.016 | 0.011 | 0.715 |
| *Paludibacteraceae* | 0.018 | 0.032 | 0.164 |
| *Gemmatimonadaceae* | 0.023 | 0.015 | 0.577 |
| *Hyphomicrobiaceae* | 0.023 | 0.010 | 0.352 |
| *Leuconostocaceae* | 0.011 | 0.001 | 0.408 |
| *unidentified_Melainabacteria* | 0.008 | 0.019 | 0.247 |
| *Xanthomonadaceae* | 0.024 | 0.005 | 0.138 |
| *Planococcaceae* | 0.006 | 0.012 | 0.552 |
| *unidentified_Rokubacteria* | 0.014 | 0.002 | 0.212 |
| *Eubacteriaceae* | 0.021 | 0.024 | 0.643 |
| *Nocardiaceae* | 0.012 | 0.003 | 0.282 |
| *Paenibacillaceae* | 0.009 | 0.002 | 0.424 |
| *Propionibacteriaceae* | 0.011 | 0.010 | 0.881 |
| *Thermomonosporaceae* | 0.007 | 0.002 | 0.474 |
| *Micromonosporaceae* | 0.014 | 0.006 | 0.281 |
| *unidentified_Cardiobacteriales* | 0.007 | 0.000 | 0.363 |
| *Methylococcaceae* | 0.007 | 0.000 | 0.363 |
| *Hymenobacteraceae* | 0.000 | 0.009 | 0.212 |
| *unidentified_Chthoniobacterales* | 0.013 | 0.004 | 0.368 |
| *unidentified_Bacillales* | 0.001 | 0.009 | 0.279 |
| *Streptosporangiaceae* | 0.007 | 0.001 | 0.399 |
| *Coxiellaceae* | 0.005 | 0.017 | 0.042 |
| *unidentified_Rickettsiales* | 0.001 | 0.011 | 0.192 |
| *Nocardiopsaceae* | 0.006 | 0.000 | 0.363 |
| *Limnochordaceae* | 0.006 | 0.000 | 0.363 |
| *Syntrophomonadaceae* | 0.017 | 0.007 | 0.115 |
| *Rhodobacteraceae* | 0.006 | 0.005 | 0.938 |
| *Campylobacteraceae* | 0.021 | 0.019 | 0.609 |
| *Chitinophagaceae* | 0.005 | 0.006 | 0.927 |
| *Corynebacteriaceae* | 0.004 | 0.012 | 0.066 |
| *unidentified_Chloroflexi* | 0.007 | 0.002 | 0.344 |
| *Microscillaceae* | 0.007 | 0.001 | 0.244 |
| *unidentified_Coriobacteriales* | 0.011 | 0.008 | 0.442 |
| *Myxococcaceae* | 0.004 | 0.000 | 0.363 |
| *Pseudonocardiaceae* | 0.005 | 0.004 | 0.908 |
| *Archangiaceae* | 0.006 | 0.001 | 0.278 |
| *unidentified_Acidobacteriales* | 0.000 | 0.009 | 0.055 |
| *Thermoactinomycetaceae* | 0.003 | 0.000 | 0.363 |
| *Methylophilaceae* | 0.003 | 0.000 | 0.363 |
| *Geminicoccaceae* | 0.005 | 0.000 | 0.105 |
| *Haliangiaceae* | 0.006 | 0.002 | 0.220 |
| *Methanobacteriaceae* | 0.000 | 0.003 | 0.275 |
| *Marinilabiliaceae* | 0.006 | 0.009 | 0.416 |
| *Oligosphaeraceae* | 0.007 | 0.005 | 0.441 |
| *unidentified_Dehalococcoidia* | 0.004 | 0.000 | 0.121 |
| *Roseiflexaceae* | 0.003 | 0.002 | 0.664 |
| *unidentified_Acidimicrobiia* | 0.001 | 0.003 | 0.252 |
| *Ktedonobacteraceae* | 0.003 | 0.001 | 0.284 |
| *Micropepsaceae* | 0.003 | 0.002 | 0.799 |
| *unidentified_Gaiellales* | 0.002 | 0.001 | 0.374 |
| *Solimonadaceae* | 0.002 | 0.000 | 0.363 |
| *Geodermatophilaceae* | 0.003 | 0.004 | 0.512 |
| *Lentimicrobiaceae* | 0.002 | 0.000 | 0.363 |
| *Woeseiaceae* | 0.002 | 0.002 | 0.817 |
| *Dietziaceae* | 0.003 | 0.004 | 0.608 |
| *unidentified_Deltaproteobacteria* | 0.003 | 0.002 | 0.825 |
| *Halomonadaceae* | 0.002 | 0.002 | 1.000 |
| *Hyphomonadaceae* | 0.002 | 0.001 | 0.309 |
| *Aerococcaceae* | 0.001 | 0.001 | 1.000 |
| *Solirubrobacteraceae* | 0.000 | 0.001 | 0.363 |
| *Iamiaceae* | 0.002 | 0.001 | 0.220 |
| *unidentified_Acidobacteriia* | 0.000 | 0.001 | 0.363 |
| *Brachyspiraceae* | 0.001 | 0.000 | 0.363 |
| *Alcanivoracaceae* | 0.001 | 0.000 | 0.363 |
| *Rhodocyclaceae* | 0.001 | 0.001 | 0.668 |
| *unidentified_Spirochaetes* | 0.001 | 0.000 | 0.363 |
| *Gemmataceae* | 0.001 | 0.000 | 0.363 |
| *Sporichthyaceae* | 0.001 | 0.000 | 0.363 |
| *Vibrionaceae* | 0.001 | 0.000 | 0.363 |
| *unidentified_Verrucomicrobiae* | 0.004 | 0.002 | 0.304 |
| *Sanguibacteraceae* | 0.000 | 0.001 | 0.363 |
| *Entomoplasmataceae* | 0.001 | 0.000 | 0.363 |
| *Acidimicrobiaceae* | 0.001 | 0.000 | 0.363 |
| *Others* | 14.727 | 12.071 | 0.312 |

**Table S3** relative abundance (%) of bacterial genera in the rectal microbiota of donkeys receiving Low-energy diet and High-energy diet.

| Taxa | Groups | | |
| --- | --- | --- | --- |
| genera | LE | HE | *P*-value |
| *Lactobacillus* | 3.624 | 3.233 | 0.904 |
| *Enterococcus* | 0.031 | 1.943 | 0.345 |
| *Pseudomonas* | 0.010 | 3.007 | 0.176 |
| *Lachnoclostridium* | 1.785 | 0.544 | 0.427 |
| *Bacteroides* | 2.463 | 2.082 | 0.823 |
| *unidentified_Bacteroidales* | 4.760 | 3.526 | 0.210 |
| *unidentified_Prevotellaceae* | 0.765 | 3.162 | 0.077 |
| *unidentified_Ruminococcaceae* | 5.687 | 4.665 | 0.064 |
| *unidentified_Lachnospiraceae* | 3.227 | 2.481 | 0.572 |
| *Cetobacterium* | 0.936 | 0.008 | 0.365 |
| *Alistipes* | 0.743 | 1.268 | 0.355 |
| *Dialister* | 0.734 | 0.004 | 0.255 |
| *unidentified_Cyanobacteria* | 0.006 | 0.655 | 0.209 |
| *unidentified_Enterobacteriaceae* | 0.043 | 0.800 | 0.181 |
| *Odoribacter* | 0.658 | 0.988 | 0.531 |
| *Blautia* | 0.202 | 0.416 | 0.478 |
| *Oscillibacter* | 0.918 | 0.863 | 0.841 |
| *Sphingomonas* | 0.049 | 0.441 | 0.170 |
| *Akkermansia* | 0.456 | 0.504 | 0.853 |
| *Alloprevotella* | 0.642 | 0.823 | 0.473 |
| *Intestinimonas* | 0.442 | 0.530 | 0.712 |
| *Ruminiclostridium* | 0.485 | 0.413 | 0.768 |
| *Clostridioides* | 0.017 | 0.226 | 0.327 |
| *Staphylococcus* | 0.009 | 0.200 | 0.365 |
| *Faecalibacterium* | 0.212 | 0.099 | 0.517 |
| *unidentified_Clostridiales* | 0.420 | 0.271 | 0.286 |
| *Coprobacillus* | 0.000 | 0.163 | 0.363 |
| *unidentified_Erysipelotrichaceae* | 0.012 | 0.176 | 0.318 |
| *Helicobacter* | 0.240 | 0.166 | 0.634 |
| *Desulfovibrio* | 0.128 | 0.373 | 0.077 |
| *Phascolarctobacterium* | 0.424 | 0.314 | 0.179 |
| *Succinivibrio* | 0.143 | 0.350 | 0.070 |
| *Candidatus_Stoquefichus* | 0.000 | 0.106 | 0.361 |
| *Klebsiella* | 0.028 | 0.161 | 0.215 |
| *Roseburia* | 0.259 | 0.140 | 0.274 |
| *Rikenella* | 0.096 | 0.153 | 0.517 |
| *unidentified_Elusimicrobia* | 0.297 | 0.077 | 0.021 |
| *Anaeroplasma* | 0.156 | 0.037 | 0.153 |
| *Bifidobacterium* | 0.092 | 0.009 | 0.291 |
| *Ileibacterium* | 0.001 | 0.074 | 0.355 |
| *Parasutterella* | 0.004 | 0.075 | 0.352 |
| *Flavobacterium* | 0.002 | 0.121 | 0.186 |
| *Agathobacter* | 0.127 | 0.042 | 0.197 |
| *Romboutsia* | 0.077 | 0.013 | 0.341 |
| *Candidatus_Soleaferrea* | 0.236 | 0.144 | 0.021 |
| *Tyzzerella* | 0.110 | 0.141 | 0.611 |
| *unidentified_Christensenellaceae* | 0.243 | 0.185 | 0.077 |
| *Parabacteroides* | 0.128 | 0.166 | 0.559 |
| *unidentified_Acidobacteria* | 0.088 | 0.027 | 0.347 |
| *Erysipelatoclostridium* | 0.015 | 0.107 | 0.203 |
| *Fibrobacter* | 0.162 | 0.211 | 0.213 |
| *Anaerovorax* | 0.237 | 0.164 | 0.017 |
| *Flavonifractor* | 0.052 | 0.029 | 0.664 |
| *Candidatus_Udaeobacter* | 0.077 | 0.015 | 0.292 |
| *Subdoligranulum* | 0.060 | 0.056 | 0.935 |
| *Muribaculum* | 0.105 | 0.058 | 0.445 |
| *Acetitomaculum* | 0.096 | 0.025 | 0.110 |
| *Polymorphobacter* | 0.000 | 0.082 | 0.175 |
| *Candidatus_Saccharimonas* | 0.054 | 0.086 | 0.450 |
| *Enterorhabdus* | 0.076 | 0.086 | 0.842 |
| *Gaiella* | 0.056 | 0.016 | 0.349 |
| *Butyricicoccus* | 0.116 | 0.084 | 0.346 |
| *Papillibacter* | 0.096 | 0.080 | 0.666 |
| *unidentified_Spirochaetaceae* | 0.076 | 0.040 | 0.235 |
| *Angelakisella* | 0.084 | 0.097 | 0.767 |
| *Dubosiella* | 0.038 | 0.002 | 0.281 |
| *Collinsella* | 0.034 | 0.001 | 0.315 |
| *Dorea* | 0.031 | 0.009 | 0.469 |
| *Faecalibaculum* | 0.001 | 0.030 | 0.370 |
| *Mucispirillum* | 0.053 | 0.056 | 0.935 |
| *Bradyrhizobium* | 0.047 | 0.027 | 0.565 |
| *Intestinibacter* | 0.033 | 0.019 | 0.635 |
| *Candidatus_Arthromitus* | 0.024 | 0.000 | 0.363 |
| *Fusobacterium* | 0.046 | 0.005 | 0.111 |
| *Elusimicrobium* | 0.069 | 0.064 | 0.820 |
| *Paraprevotella* | 0.037 | 0.027 | 0.727 |
| *Negativibacillus* | 0.046 | 0.053 | 0.775 |
| *Allobaculum* | 0.000 | 0.021 | 0.363 |
| *Methylobacterium* | 0.007 | 0.037 | 0.200 |
| *Aeromonas* | 0.021 | 0.000 | 0.363 |
| *unidentified_Rhizobiaceae* | 0.004 | 0.035 | 0.197 |
| *Frigoribacterium* | 0.003 | 0.034 | 0.196 |
| *Marvinbryantia* | 0.079 | 0.047 | 0.061 |
| *Streptococcus* | 0.081 | 0.063 | 0.145 |
| *Saccharofermentans* | 0.063 | 0.048 | 0.351 |
| *Massilia* | 0.007 | 0.035 | 0.275 |
| *Delftia* | 0.000 | 0.018 | 0.349 |
| *Oribacterium* | 0.060 | 0.044 | 0.258 |
| *Mycoplasma* | 0.018 | 0.004 | 0.414 |
| *Luteibacter* | 0.016 | 0.007 | 0.587 |
| *Rhodoferax* | 0.001 | 0.028 | 0.184 |
| *Acidothermus* | 0.019 | 0.003 | 0.340 |
| *Bacillus* | 0.013 | 0.018 | 0.797 |
| *Oceanobacillus* | 0.016 | 0.002 | 0.374 |
| *Streptomyces* | 0.020 | 0.006 | 0.353 |
| *Anaerotruncus* | 0.017 | 0.022 | 0.805 |
| *Dyadobacter* | 0.001 | 0.023 | 0.191 |
| *unidentified_Rhizobiales* | 0.023 | 0.015 | 0.691 |
| *Ralstonia* | 0.017 | 0.000 | 0.253 |
| *Pedobacter* | 0.002 | 0.021 | 0.217 |
| *Cloacibacillus* | 0.013 | 0.003 | 0.466 |
| *Flexilinea* | 0.022 | 0.033 | 0.329 |
| *Acetobacter* | 0.012 | 0.000 | 0.363 |
| *Arthrobacter* | 0.021 | 0.016 | 0.720 |
| *Mycobacterium* | 0.021 | 0.003 | 0.239 |
| *Jatrophihabitans* | 0.016 | 0.011 | 0.715 |
| *Lachnospira* | 0.032 | 0.026 | 0.570 |
| *Weissella* | 0.011 | 0.001 | 0.408 |
| *Sutterella* | 0.001 | 0.012 | 0.337 |
| *Mogibacterium* | 0.043 | 0.028 | 0.106 |
| *Variovorax* | 0.002 | 0.017 | 0.234 |
| *Pedomicrobium* | 0.018 | 0.009 | 0.433 |
| *Acinetobacter* | 0.011 | 0.012 | 0.967 |
| *Brevundimonas* | 0.001 | 0.015 | 0.170 |
| *unidentified_Melainabacteria* | 0.008 | 0.019 | 0.247 |
| *Candidatus_Solibacter* | 0.014 | 0.004 | 0.339 |
| *Sphingopyxis* | 0.010 | 0.004 | 0.594 |
| *Planomicrobium* | 0.000 | 0.009 | 0.363 |
| *Harryflintia* | 0.019 | 0.025 | 0.468 |
| *Proteocatella* | 0.009 | 0.000 | 0.363 |
| *Pseudoxanthomonas* | 0.010 | 0.001 | 0.359 |
| *Mucilaginibacter* | 0.000 | 0.009 | 0.334 |
| *Bilophila* | 0.021 | 0.022 | 0.921 |
| *unidentified_Beijerinckiaceae* | 0.000 | 0.018 | 0.123 |
| *unidentified_Rokubacteria* | 0.014 | 0.002 | 0.212 |
| *Sphaerochaeta* | 0.026 | 0.011 | 0.044 |
| *Rhodococcus* | 0.012 | 0.003 | 0.282 |
| *Psychrobacter* | 0.009 | 0.001 | 0.359 |
| *Microlunatus* | 0.009 | 0.008 | 0.923 |
| *Pseudobutyrivibrio* | 0.030 | 0.015 | 0.032 |
| *Actinomadura* | 0.007 | 0.002 | 0.474 |
| *Ignatzschineria* | 0.007 | 0.000 | 0.363 |
| *Anaerovibrio* | 0.026 | 0.023 | 0.658 |
| *Exiguobacterium* | 0.001 | 0.007 | 0.358 |
| *Duganella* | 0.000 | 0.013 | 0.175 |
| *Acetatifactor* | 0.015 | 0.001 | 0.071 |
| *Sphaerisporangium* | 0.007 | 0.001 | 0.399 |
| *Candidatus_Xiphinematobacter* | 0.013 | 0.004 | 0.368 |
| *Hymenobacter* | 0.000 | 0.009 | 0.212 |
| *Holdemanella* | 0.006 | 0.001 | 0.402 |
| *unidentified_Rickettsiales* | 0.001 | 0.011 | 0.192 |
| *Coxiella* | 0.005 | 0.017 | 0.042 |
| *Thermobifida* | 0.006 | 0.000 | 0.363 |
| *Pseudaminobacter* | 0.014 | 0.002 | 0.163 |
| *Lysobacter* | 0.011 | 0.002 | 0.268 |
| *Anaerosporobacter* | 0.010 | 0.000 | 0.093 |
| *Rhodopseudomonas* | 0.003 | 0.010 | 0.303 |
| *Frondihabitans* | 0.000 | 0.005 | 0.363 |
| *Sphingoaurantiacus* | 0.000 | 0.008 | 0.185 |
| *Caproiciproducens* | 0.005 | 0.012 | 0.181 |
| *Bryobacter* | 0.007 | 0.003 | 0.530 |
| *Dongia* | 0.009 | 0.003 | 0.349 |
| *Acidiphilium* | 0.000 | 0.009 | 0.176 |
| *Devosia* | 0.003 | 0.009 | 0.323 |
| *Mailhella* | 0.017 | 0.005 | 0.012 |
| *Sinibacillus* | 0.005 | 0.000 | 0.363 |
| *Campylobacter* | 0.021 | 0.019 | 0.609 |
| *Shuttleworthia* | 0.003 | 0.013 | 0.067 |
| *Turicibacter* | 0.006 | 0.001 | 0.361 |
| *unidentified_Chloroflexi* | 0.007 | 0.002 | 0.344 |
| *Catenisphaera* | 0.014 | 0.014 | 1.000 |
| *Butyricimonas* | 0.012 | 0.007 | 0.315 |
| *unidentified_Corynebacteriaceae* | 0.002 | 0.005 | 0.490 |
| *Stenotrophobacter* | 0.005 | 0.001 | 0.364 |
| *Acidibacter* | 0.003 | 0.005 | 0.662 |
| *Prevotella* | 0.000 | 0.004 | 0.363 |
| *Reyranella* | 0.008 | 0.003 | 0.427 |
| *Novosphingobium* | 0.002 | 0.006 | 0.354 |
| *Faecalitalea* | 0.002 | 0.005 | 0.518 |
| *unidentified_Burkholderiaceae* | 0.006 | 0.008 | 0.691 |
| *Chryseolinea* | 0.004 | 0.000 | 0.363 |
| *Anaeromyxobacter* | 0.006 | 0.001 | 0.278 |
| *unidentified_Gammaproteobacteria* | 0.005 | 0.004 | 0.830 |
| *Anaerofustis* | 0.013 | 0.011 | 0.597 |
| *Phoenicibacter* | 0.011 | 0.008 | 0.442 |
| *Oxalobacter* | 0.006 | 0.004 | 0.771 |
| *Kocuria* | 0.006 | 0.002 | 0.305 |
| *unidentified_Alphaproteobacteria* | 0.009 | 0.002 | 0.174 |
| *Bosea* | 0.001 | 0.004 | 0.294 |
| *Gallicola* | 0.003 | 0.000 | 0.363 |
| *Caulobacter* | 0.002 | 0.005 | 0.487 |
| *Oscillospira* | 0.002 | 0.010 | 0.017 |
| *Peptococcus* | 0.006 | 0.005 | 0.907 |
| *Methylorosula* | 0.001 | 0.008 | 0.098 |
| *Sporosarcina* | 0.003 | 0.000 | 0.363 |
| *Terriglobus* | 0.000 | 0.006 | 0.120 |
| *Ammoniibacillus* | 0.003 | 0.000 | 0.363 |
| *Novibacillus* | 0.003 | 0.000 | 0.363 |
| *Rhizobacter* | 0.004 | 0.006 | 0.731 |
| *Pseudochrobactrum* | 0.000 | 0.006 | 0.177 |
| *Hyphomicrobium* | 0.005 | 0.002 | 0.383 |
| *Candidatus_Alysiosphaera* | 0.005 | 0.000 | 0.105 |
| *Pseudonocardia* | 0.005 | 0.003 | 0.536 |
| *Aureimonas* | 0.001 | 0.005 | 0.292 |
| *Fournierella* | 0.003 | 0.007 | 0.228 |
| *Haliangium* | 0.006 | 0.002 | 0.220 |
| *Paracoccus* | 0.003 | 0.003 | 1.000 |
| *Rhodoplanes* | 0.005 | 0.003 | 0.674 |
| *Gemmatimonas* | 0.003 | 0.002 | 0.863 |
| *Acidovorax* | 0.006 | 0.005 | 0.796 |
| *Sphingobium* | 0.003 | 0.001 | 0.596 |
| *Pyramidobacter* | 0.005 | 0.004 | 0.740 |
| *Parvibaculum* | 0.003 | 0.000 | 0.363 |
| *Methanobrevibacter* | 0.000 | 0.003 | 0.275 |
| *Methylophilus* | 0.003 | 0.000 | 0.363 |
| *Phenylobacterium* | 0.000 | 0.004 | 0.191 |
| *unidentified_Dehalococcoidia* | 0.004 | 0.000 | 0.121 |
| *Serratia* | 0.001 | 0.003 | 0.284 |
| *Hydrogenoanaerobacterium* | 0.002 | 0.002 | 0.830 |
| *Mesorhizobium* | 0.003 | 0.004 | 0.576 |
| *unidentified_Acidimicrobiia* | 0.001 | 0.003 | 0.252 |
| *Enhydrobacter* | 0.001 | 0.003 | 0.252 |
| *Amaricoccus* | 0.002 | 0.000 | 0.363 |
| *Catabacter* | 0.000 | 0.003 | 0.259 |
| *Lawsonia* | 0.002 | 0.000 | 0.363 |
| *Cutibacterium* | 0.002 | 0.002 | 0.820 |
| *Cuneatibacter* | 0.002 | 0.000 | 0.363 |
| *Arenimonas* | 0.004 | 0.002 | 0.577 |
| *Rickettsiella* | 0.000 | 0.003 | 0.185 |
| *Lentimicrobium* | 0.002 | 0.000 | 0.363 |
| *Halomonas* | 0.002 | 0.002 | 1.000 |
| *Megamonas* | 0.003 | 0.001 | 0.252 |
| *Garciella* | 0.002 | 0.000 | 0.363 |
| *unidentified_Deltaproteobacteria* | 0.003 | 0.002 | 0.825 |
| *Amycolatopsis* | 0.000 | 0.002 | 0.363 |
| *Oligella* | 0.003 | 0.002 | 0.781 |
| *Thermobacillus* | 0.002 | 0.000 | 0.363 |
| *Ammoniphilus* | 0.001 | 0.002 | 0.590 |
| *Synergistes* | 0.000 | 0.002 | 0.235 |
| *Panacagrimonas* | 0.002 | 0.000 | 0.363 |
| *Ezakiella* | 0.002 | 0.000 | 0.363 |
| *Kurthia* | 0.002 | 0.000 | 0.363 |
| *Woeseia* | 0.002 | 0.000 | 0.363 |
| *Gemmatirosa* | 0.000 | 0.002 | 0.235 |
| *Microvirga* | 0.002 | 0.001 | 0.550 |
| *Mucinivorans* | 0.006 | 0.001 | 0.002 |
| *Barnesiella* | 0.002 | 0.000 | 0.363 |
| *unidentified_Gaiellales* | 0.002 | 0.001 | 0.374 |
| *Altererythrobacter* | 0.003 | 0.000 | 0.111 |
| *Gordonibacter* | 0.000 | 0.002 | 0.363 |
| *Dietzia* | 0.003 | 0.004 | 0.608 |
| *Anaerostipes* | 0.001 | 0.002 | 0.788 |
| *Eubacterium* | 0.006 | 0.003 | 0.196 |
| *Lactococcus* | 0.002 | 0.001 | 0.550 |
| *Paenibacillus* | 0.002 | 0.000 | 0.363 |
| *Sediminispirochaeta* | 0.002 | 0.003 | 0.341 |
| *Herbaspirillum* | 0.001 | 0.000 | 0.363 |
| *Dokdonella* | 0.000 | 0.001 | 0.363 |
| *Leisingera* | 0.001 | 0.000 | 0.363 |
| *Rummeliibacillus* | 0.001 | 0.003 | 0.361 |
| *unidentified_Acidobacteriia* | 0.000 | 0.001 | 0.363 |
| *Sanguibacter* | 0.000 | 0.001 | 0.363 |
| *unidentified_Verrucomicrobiae* | 0.004 | 0.002 | 0.304 |
| *Labrys* | 0.003 | 0.002 | 0.756 |
| *Aerosphaera* | 0.001 | 0.000 | 0.363 |
| *Terrimonas* | 0.001 | 0.001 | 0.668 |
| *Iamia* | 0.002 | 0.001 | 0.220 |
| *Spirosoma* | 0.000 | 0.001 | 0.363 |
| *Georgfuchsia* | 0.001 | 0.001 | 0.668 |
| *Veillonella* | 0.001 | 0.002 | 0.688 |
| *Aerococcus* | 0.000 | 0.001 | 0.363 |
| *Kaistia* | 0.000 | 0.002 | 0.203 |
| *Erysipelothrix* | 0.001 | 0.000 | 0.363 |
| *Conexibacter* | 0.000 | 0.001 | 0.363 |
| *unidentified_Spirochaetes* | 0.001 | 0.000 | 0.363 |
| *Alcanivorax* | 0.001 | 0.000 | 0.363 |
| *Blastocatella* | 0.000 | 0.002 | 0.203 |
| *Ruminobacter* | 0.001 | 0.002 | 0.220 |
| *Vibrio* | 0.001 | 0.000 | 0.363 |
| *Gemella* | 0.001 | 0.002 | 0.409 |
| *Brachyspira* | 0.001 | 0.000 | 0.363 |
| *Corynebacterium* | 0.000 | 0.001 | 0.363 |
| *Bauldia* | 0.002 | 0.000 | 0.203 |
| *Candidatus_Tammella* | 0.001 | 0.001 | 1.000 |
| *unidentified_Bacteria* | 0.002 | 0.002 | 0.688 |
| *Rothia* | 0.001 | 0.001 | 0.668 |
| *Entomoplasma* | 0.001 | 0.000 | 0.363 |
| *Breznakia* | 0.000 | 0.001 | 0.363 |
| *Holdemania* | 0.000 | 0.001 | 0.175 |
| *Cellulosilyticum* | 0.001 | 0.001 | 1.000 |
| *Planosporangium* | 0.001 | 0.000 | 0.363 |
| *Longispora* | 0.001 | 0.000 | 0.363 |
| *Others* | 64.573 | 60.800 | 0.384 |

***The rectum metabolome determination by liquid chromatography-mass spectrometry（LC-MS）***

**1. Metabolites Extraction**

Collected rectum digesta (100 mg) were individually grounded with liquid nitrogen and the homogenate was resuspended with prechilled 80% methanol and 0.1% formic acid by well vortexing. The samples were incubated on ice for 5 min and then were centrifuged at 15000 rpm, 4°C for 5 min. A some of supernatant was diluted to final concentration containing 53% methanol by LC-MS grade water. The samples were subsequently transferred to a fresh Eppendorf tube and then were centrifuged at 15000 g, 4°C for 10 min. Finally, the supernatant was injected into the LC-MS/MS system analysis.

**2. UHPLC-MS/MS Analysis**

LC-MS/MS analyses were performed using a Vanquish UHPLC system (Thermo Fisher) coupled with an Orbitrap Q Exactive series mass spectrometer (Thermo Fisher). Samples were injected onto an Hyperil Gold column (100×2.1 mm, 1.9μm) using a 16- min linear gradient at a flow rate of 0.2mL/min. The eluents for the positive polarity mode were eluent A (0.1% FA in Water) and eluent B (Methanol).The eluents for the negative polarity mode were eluent A (5 mM ammonium acetate, pH 9.0) and eluent B (Methanol).The solvent gradient was set as follows: 2% B, 1.5 min; 2-100% B, 12.0 min; 100% B, 14.0 min；100-2% B, 14.1 min；2% B, 17 min. Q Exactive series mass spectrometer was operated in positive/negative polarity mode with spray voltage of 3.2 kV, capillary temperature of 320°C, sheath gas flow rate of 35 arb and aux gas flow rate of 10 arb.

**3. Database search**

The raw data files generated by UHPLC-MS/MS were processed using the Compound Discoverer 3.1 (CD3.1, Thermo Fisher) to perform peak alignment, peak picking, and quantitation for each metabolite. The main parameters were set as follows: retention time tolerance, 0.2 minutes; actual mass tolerance, 5ppm; signal intensity tolerance, 30%; signal/noise ratio, 3; and minimum intensity, 100000. After that, peak intensities were normalized to the total spectral intensity. The normalized data was used to predict the molecular formula based on additive ions, molecular ion peaks and fragment ions. And then peaks were matched with the mzCloud (https://www.mzcloud.org/), mzVault and Mass List database to obtained the accurate qualitative and relative quantitative results.

**Table S4** Different metabolites between HE and LE groups with LC-MS/MS (ESI+)

| Name | log2FC | P-value | VIP | Up or Down |
| --- | --- | --- | --- | --- |
| 2,4-dihydroxyheptadec-16-en-1-yl acetate | 1.603 | <0.01 | 1.965 | up |
| Fumonisin B1 | -2.528 | <0.01 | 3.118 | down |
| Fumonisin B2 | -2.670 | <0.01 | 3.313 | down |
| D-(+)-Camphor | 1.199 | <0.01 | 1.464 | up |
| Fenvalerate | -2.352 | <0.01 | 2.901 | down |
| N-Acetyl-5-aminosalicylic acid | -4.332 | <0.01 | 5.133 | down |
| LPE 16:2 | -3.059 | <0.01 | 3.660 | down |
| Cynaropicrin | 1.398 | <0.01 | 1.686 | up |
| Desthiobiotin | 1.921 | <0.01 | 2.424 | up |
| N-(9-oxodecyl)acetamide | 1.124 | <0.01 | 1.392 | up |
| N6,N6,N6-Trimethyl-L-lysine | -1.652 | <0.01 | 2.091 | down |
| Histamine | -3.529 | <0.01 | 4.237 | down |
| Phosphocreatine | -1.056 | <0.01 | 1.278 | down |
| N-Cyclohexyl-N-methylcyclohexanamine | -3.723 | <0.01 | 4.276 | down |
| Vindoline | -1.983 | <0.01 | 2.515 | down |
| 2-phenyl-4H-furo[2,3-h]chromen-4-one | -1.118 | <0.01 | 1.358 | down |
| N-Methyl-L-arginine hydrochloride | -1.114 | <0.01 | 1.378 | down |
| Ethyl oleate | 1.085 | <0.01 | 1.353 | up |
| Phenylpyruvic Acid | 1.194 | <0.01 | 1.504 | up |
| Isohomovanillic acid | 1.114 | <0.01 | 1.406 | up |
| 2-Arachidonoyl glycerol | 1.028 | <0.01 | 1.240 | up |
| cis-gondoic acid | -1.250 | <0.01 | 1.491 | down |
| 15-Deoxy-Δ12,14-prostaglandin A1 | 1.272 | <0.01 | 1.508 | up |
| 1,5-Diaminopentane | -2.195 | <0.01 | 2.764 | down |
| Methionine | -1.684 | <0.01 | 1.983 | down |
| 4-(allyloxy)-1,2-dihydroquinolin-2-one | 1.234 | <0.01 | 1.542 | up |
| 6-Pentyl-2H-pyran-2-one | 1.004 | 0.001 | 1.193 | up |
| L-(+)-Citrulline | -1.198 | 0.001 | 1.414 | down |
| DL-Lysine | -1.065 | 0.001 | 1.261 | down |
| SDMA/ADMA | -1.271 | 0.001 | 1.498 | down |
| (R)-Lipoic Acid | -1.287 | 0.001 | 1.526 | down |
| Asp-Phe methyl ester | -1.100 | 0.001 | 1.331 | down |
| 17α-Ethinylestradiol | 1.316 | 0.001 | 1.578 | up |
| Serotonin | -1.119 | 0.002 | 1.410 | down |
| Isomaltose | 1.135 | 0.002 | 1.313 | up |
| L-Aspartic acid β-benzyl ester | 1.262 | 0.002 | 1.478 | up |
| Lysope 14:0 | 1.147 | 0.002 | 1.392 | up |
| Dl-Indole-3-lactic acid | -1.567 | 0.002 | 1.778 | down |
| Menaquinone | 1.144 | 0.003 | 1.349 | up |
| Lysopc 15:0 | -1.298 | 0.003 | 1.580 | down |
| Nervonic ceramide | -1.567 | 0.003 | 1.761 | down |
| Methyl N-cyano-N'-[2-(2,2-dichlorocyclopropyl)ethyl]carbamimidothioate | -1.244 | 0.003 | 1.590 | down |
| Kahweol | 1.647 | 0.004 | 1.899 | up |
| Lagochilin | 1.265 | 0.004 | 1.448 | up |
| L-Isoleucine | -1.038 | 0.005 | 1.190 | down |
| Acetylcholine | -1.366 | 0.006 | 1.526 | down |
| XLR11 N-(4-hydroxypentyl) metabolite | 1.112 | 0.007 | 1.539 | up |
| D-Phenylalanine | -1.116 | 0.009 | 1.260 | down |
| 5α-Androstan-3,6,17-trione | 1.124 | 0.009 | 1.557 | up |
| Butein | 1.163 | 0.011 | 1.304 | up |
| Indole | -1.003 | 0.011 | 1.356 | down |
| gamma-Glutamylmethionine | -1.318 | 0.011 | 1.609 | down |
| Glutamine | -1.183 | 0.012 | 1.391 | down |
| Argininosuccinic acid | 1.309 | 0.014 | 1.426 | up |
| N-[1-(4-methoxy-2-oxo-2H-pyran-6-yl)-2-methylbutyl]acetamide | 1.482 | 0.014 | 2.387 | up |
| N-[2-(1,5-dimethyl-4-nitro-1H-pyrazol-3-yl)vinyl]-N,N-dimethylamine | -4.067 | 0.018 | 3.789 | down |
| Cholest-4-en-3-one | 5.575 | 0.019 | 4.503 | up |
| α-Aspartylphenylalanine | -1.245 | 0.020 | 1.654 | down |
| 3-hydroxy-2-octylpentanedioic acid | -2.304 | 0.025 | 2.271 | down |
| Hypoxanthine | -1.330 | 0.027 | 1.431 | down |
| Adenine | -1.114 | 0.029 | 1.248 | down |
| Tyramine | -1.962 | 0.029 | 2.019 | down |
| L-Phenylalanine | -1.042 | 0.029 | 1.172 | down |
| 5-Hydroxyindole-3-acetic acid | -5.098 | 0.031 | 4.162 | down |
| LPE 14:0 | 1.172 | 0.031 | 1.206 | up |
| 8-Hydroxyquinoline | -2.965 | 0.032 | 2.651 | down |
| DL-Arginine | -1.673 | 0.032 | 1.844 | down |
| L(-)-Carnitine | -1.032 | 0.035 | 1.441 | down |
| 5-methyl-3-(2-morpholino-2-oxoethyl)-1,3-benzoxazol-2(3H)-one | 1.785 | 0.039 | 1.697 | up |
| Etiocholanolone | -2.035 | 0.040 | 1.874 | down |

**Table S5**  Different metabolites between HE and LE groups with LC-MS/MS (ESI-)

| Name | log2FC | Pvalue | VIP | Up.Down |
| --- | --- | --- | --- | --- |
| Saccharin | -2.187 | <0.01 | 2.447 | down |
| Cyclamic acid | -1.951 | <0.01 | 2.200 | down |
| Trehalose | 2.391 | <0.01 | 2.675 | up |
| Asp-glu | -1.797 | <0.01 | 1.998 | down |
| γ-Glutamylglutamic acid | -1.380 | <0.01 | 1.527 | down |
| Glycerol-3-phosphate | -1.469 | <0.01 | 1.612 | down |
| Acetildenafil | -2.214 | <0.01 | 2.547 | down |
| D-Methionine | -1.556 | <0.01 | 1.726 | down |
| L-Aspartic acid | -1.746 | <0.01 | 1.928 | down |
| Mycophenolic acid | -1.527 | <0.01 | 1.725 | down |
| D-Galactosamine | -1.316 | <0.01 | 1.444 | down |
| Calcitriol | -2.587 | <0.01 | 2.879 | down |
| N-Acetylaspartic acid | -1.857 | <0.01 | 2.014 | down |
| N-Methylthreonine | -1.331 | <0.01 | 1.468 | down |
| 7-Ketodeoxycholic acid | -1.783 | <0.01 | 2.045 | down |
| Pyrophosphate | -1.935 | <0.01 | 2.269 | down |
| N-Methyl-a-aminoisobutyric acid | -1.434 | 0.001 | 1.550 | down |
| 11β-Prostaglandin F2α | -1.239 | 0.001 | 1.423 | down |
| Protocatechuic Aldehyde | 1.099 | 0.001 | 1.327 | up |
| Isoleucine | -1.456 | 0.001 | 1.552 | down |
| 20-Hydroxy-(5Z,8Z,11Z,14Z)-eicosatetraenoic acid | -1.570 | 0.001 | 1.848 | down |
| Ornithine | -1.805 | 0.001 | 1.872 | down |
| Ofloxacin impurity E | -1.456 | 0.001 | 1.705 | down |
| Citrulline | -1.300 | 0.001 | 1.361 | down |
| 17-α-Methyltestosterone | -2.151 | 0.001 | 2.254 | down |
| FAHFA (2:0/21:0) | -1.418 | 0.002 | 1.618 | down |
| FAHFA (4:0/18:0) | -1.309 | 0.002 | 1.444 | down |
| 4-Hexylresorcinol | 1.526 | 0.002 | 1.624 | up |
| Lysine | -1.094 | 0.003 | 1.226 | down |
| Anacardic acid | -3.357 | 0.003 | 3.277 | down |
| Lysopc 18:3 | 1.367 | 0.003 | 1.543 | up |
| Urethane | -1.098 | 0.003 | 1.268 | down |
| methyl 5-{[2-(ethoxycarbonyl)-3-oxohex-1-enyl]amino}-2-furoate | -1.553 | 0.004 | 1.731 | down |
| N1-(3-amino-4-chlorophenyl)-2-[2,4-di(tert-pentyl)phenoxy]acetamide | -1.307 | 0.004 | 1.497 | down |
| Cholic acid | -1.863 | 0.005 | 2.046 | down |
| L-Serine | -1.265 | 0.005 | 1.277 | down |
| L-Glutamine | -1.521 | 0.005 | 1.628 | down |
| Flavin adenine dinucleotide | -1.073 | 0.005 | 1.186 | down |
| Flavin mononucleotide (FMN) | -1.242 | 0.006 | 1.396 | down |
| Hesperetin | 1.658 | 0.006 | 1.980 | up |
| Corchorifatty acid F | 1.131 | 0.006 | 1.367 | up |
| 2-Hydroxycaproic acid | -1.699 | 0.006 | 1.660 | down |
| FAHFA (18:0/22:3) | -1.276 | 0.006 | 1.313 | down |
| Nicotinic Acid | -1.171 | 0.007 | 1.251 | down |
| DL-α-Methoxyphenylacetic acid | -1.390 | 0.008 | 1.797 | down |
| Orotidine | -1.575 | 0.008 | 1.672 | down |
| 3-Hydroxy-3-Methyl Butyric Acid | -1.551 | 0.009 | 1.524 | down |
| LPS 20:1 | -1.657 | 0.009 | 1.937 | down |
| Deoxyadenosine | -2.350 | 0.010 | 2.105 | down |
| Trolox | -1.086 | 0.012 | 1.175 | down |
| UDP-N-acetylglucosamine | -1.071 | 0.013 | 1.145 | down |
| Uridine5-diphosphate | -1.085 | 0.013 | 1.116 | down |
| LPG 19:1 | -1.614 | 0.014 | 1.903 | down |
| Butylparaben | 1.056 | 0.016 | 1.556 | up |
| Dl-Tropic acid | -1.142 | 0.017 | 1.544 | down |
| Thymidine | -1.270 | 0.021 | 1.378 | down |
| 2-(Formylamino)Benzoic Acid | -1.350 | 0.022 | 1.400 | down |
| cAMP | -1.422 | 0.025 | 1.328 | down |
| D-glyceraldehdye-3-phosphate | -1.080 | 0.026 | 1.225 | down |
| Adenosine 3'5'-cyclic monophosphate | -1.007 | 0.027 | 1.164 | down |
| 2'-Deoxyinosine | -1.527 | 0.033 | 1.436 | down |
| Methyl indole-3-acetate | -1.265 | 0.034 | 1.284 | down |
| Deoxyribose 5-Phosphate | -1.746 | 0.034 | 1.648 | down |
| Tetradecanedioic acid | -1.184 | 0.044 | 1.479 | down |
| LPE 16:1 | -1.127 | 0.045 | 1.062 | down |
| D-Mannose 6-phosphate | -1.071 | 0.046 | 1.114 | down |
| 2-(acetylamino)-4-(methylthio)butanoic acid | -1.269 | 0.047 | 1.250 | down |

[1] X. Liu, H. Fan, X. Ding, Z. Hong, Y. Nei, Z. Liu, G. Li, and H. Guo, Analysis of the gut microbiota by high-throughput sequencing of the V5-V6 regions of the 16S rRNA gene in donkey. Curr. Microbiol. 68 (2014) 657-62.
